# Efficacy and Safety of Tenofovir Alafenamide (TAF) and Tenofovir Disoproxil Fumarate (TDF) Followed by TAF in Chronic Hepatitis B Patients of East Asian Ethnicity Following 5 Years of Treatment

**DOI:** 10.1111/apt.70327

**Published:** 2025-08-12

**Authors:** Grace Lai‐Hung Wong, Edward Gane, Calvin Q. Pan, Scott Fung, Mang M. Ma, Namiki Izumi, Shalimar, Seng Gee Lim, Wan‐Long Chuang, Rajiv Mehta, Young‐Suk Lim, Leland J. Yee, John F. Flaherty, Frida Abramov, Hongyuan Wang, Maria Buti

**Affiliations:** ^1^ Department of Medicine and Therapeutics, Medical Data Analytics Centre (MDAC) The Chinese University of Hong Kong Hong Kong SAR China; ^2^ Faculty of Medicine University of Auckland Auckland New Zealand; ^3^ Division of Hepatology, Department of Medicine, Guangzhou Eighth People's Hospital Guangzhou Medical University Guangzhou China; ^4^ Division of Gastroenterology and Hepatology, Department of Medicine, NYU Langone Health New York University Grossman School of Medicine New York City New York USA; ^5^ Department of Medicine, Toronto Center for Liver Disease University of Toronto Toronto Ontario Canada; ^6^ University of Alberta Edmonton Alberta Canada; ^7^ Department of Gastroenterology and Hepatology Musashino Red Cross Hospital Tokyo Japan; ^8^ Department of Gastroenterology and Human Nutrition Unit All India Institute of Medical Sciences New Delhi India; ^9^ Division of Gastroenterology and Hepatology, Department of Medicine National University Hospital Singapore; ^10^ Hepatobiliary Division, Department of Internal Medicine and Hepatitis Research Center, Kaohsiung Medical University Hospital Kaohsiung Medical University Kaohsiung Taiwan; ^11^ School of Medicine and Hepatitis Research Center, College of Medicine, Center for Cancer Research and Center for Liquid Biopsy Kaohsiung Medical University Kaohsiung Taiwan; ^12^ Liver Clinic SIDS Hospital Surat India; ^13^ Department of Gastroenterology, Asan Medical Center University of Ulsan College of Medicine Seoul South Korea; ^14^ Gilead Sciences Foster City California USA; ^15^ Hospital Universitario Vall d'Hebron and CIBEREHD del Instituto Carlos III Barcelona Spain

**Keywords:** ALT normalisation, hepatitis B, tenofovir alafenamide, tenofovir disoproxil fumarate

## Abstract

**Background:**

Tenofovir alafenamide (TAF) has shown non‐inferior efficacy to tenofovir disoproxil fumarate (TDF), with superior bone and renal safety.

**Aim:**

To characterise 5‐year TAF efficacy and safety in patients of East Asian ethnicity from pivotal Phase 3 studies.

**Methods:**

Patients were randomised (2:1) to receive TAF or TDF for up to 3 years of double‐blind treatment, followed by open‐label TAF. Patients either continued TAF or switched from TDF to TAF at Week 96 (TDF → TAF 3 years) or Week 144 (TDF → TAF 2 years) of treatment. Efficacy endpoints (virologic, biochemical and serologic) and safety were assessed.

**Results:**

Among 591 patients of East Asian ethnicity (TAF, *n* = 401; TDF → TAF 3 years, *n* = 84; TDF → TAF 2 years, *n* = 106), high rates of virologic control were achieved (89%, 94% and 92%, respectively) at Year 5 (missing = failure analysis). At Year 5, rates of alanine aminotransferase normalisation (85%, 90% and 78%) and hepatitis B e antigen loss (36%, 43% and 44%) were similar. Following the switch from TDF to TAF, changes in fasting lipid parameters were consistent with removal of the known lipid‐lowering effect of TDF. However, changes in the total cholesterol to high‐density lipoprotein ratio (marker of cardiovascular risk) were minimal and comparable in all groups by Year 5. Renal and bone parameters improved after switching.

**Conclusions:**

Through 5 years, rates of virologic suppression were high in East Asian patients treated with TAF or switched from TDF to TAF. TAF and TDF were well tolerated, with improved renal and bone safety observed in patients switching from TDF to TAF.

## Graphical abstract

In East Asian patients with chronic hepatitis B, rates of virologic suppression through 5 years were high in those treated continuously with tenofovir alafenamide (TAF) and in those switched from tenofovir disoproxil fumarate (TDF) to TAF. Improved renal and bone safety was observed in patients switching from TDF to TAF.

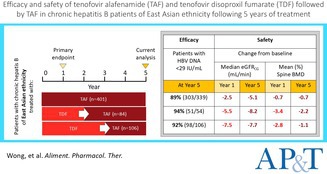

## 1 Introduction

Hepatitis B virus (HBV) infection remains a major global health concern, with an estimated 254 million infected in 2022 and over 1 million annual deaths, primarily due to cirrhosis and hepatocellular carcinoma (HCC) [1]. Long‐term antiviral treatment is essential to suppress viral replication, reduce the risk of liver‐related complications and improve survival [2, 3, 4].

Preferred antiviral treatments for chronic hepatitis B (CHB) are oral nucleos(t)ide analogues (NAs) such as entecavir (ETV) and the tenofovir (TFV) prodrugs, tenofovir disoproxil fumarate (TDF) and tenofovir alafenamide (TAF) [2, 3, 4]. Both prodrugs inhibit the HBV reverse transcriptase/polymerase to suppress viral replication, but their safety profiles differ [2, 3, 4]. Long‐term TDF use achieves durable viral suppression with no evidence of antiviral resistance development [5, 6, 7, 8], though some patients experience bone and renal toxicity [6, 9, 10]. TAF, a newer TFV prodrug, was designed to enable enhanced delivery of the active form (TFV‐diphosphate) to hepatocytes, with reduced systemic TFV exposure relative to TDF when given at approved doses [11, 12, 13]. In two multinational Phase 3 studies in viraemic patients with CHB, TAF demonstrated non‐inferior antiviral efficacy, higher rates of alanine aminotransferase (ALT) normalisation and superior bone and renal safety compared with TDF at Weeks 48 and 96 [14, 15]. In these two studies of hepatitis B e antigen (HBeAg)‐negative [14] and HBeAg‐positive [15] patients, all patients were to receive open‐label TAF following double‐blind treatment through Year 8, with 5‐year results recently published [16]. In addition, 5‐year data have now been published for a separate cohort of 334 patients enrolled into these two studies in China, which led to local registration [17]. Additional real‐world studies have also shown that switching virally suppressed CHB patients from TDF to TAF treatment maintains high levels of viral suppression and improves bone mineral density (BMD) and estimated glomerular filtration rate by Cockcroft−Gault (eGFR_CG_) [18, 19, 20, 21, 22]. Based on these results, treatment guidelines now recommend TAF or ETV over TDF for CHB patients at risk of bone or renal toxicity [3, 4].

Given the high burden of HBV in East Asian populations, both in countries within the region and in individuals of East Asian descent globally, the associated morbidity and mortality of HBV‐related liver disease in this population warrants focused attention. Additionally, Asian patients with CHB, particularly those with lower body mass index (BMI), may be at higher risk for osteoporosis and other bone complications. Therefore, it is important to evaluate long‐term treatment outcomes in this group [23, 24]. This post hoc analysis aimed to characterise the 5‐year efficacy and safety of TAF in patients of East Asian ethnicity enrolled in two global Phase 3 studies, including those treated continuously with TAF and those switched from TDF after up to 3 years. The goal was to provide additional evidence to support clinical decision‐making for healthcare providers managing CHB in East Asian populations. In the parent global studies 108 and 110, [14, 15] 247 (58%) and 441 (51%) patients, respectively, were of non‐East Asian ethnicity. Here, therefore, we provide a more focused analysis of this patient population [14, 15].

## 2 Methods

### 2.1 Study Design

This was a post hoc analysis of East Asian patients included in two Phase 3, randomised, double‐blind, active‐controlled studies performed in patients with HBeAg‐negative (study GS‐US‐320‐0108; NCT01940341 [Study 108]) [14] or HBeAg‐positive (study GS‐US‐320‐0110; NCT01940471 [Study 110]) [15] CHB. Full details of the design and methods of these studies have been published previously [14, 15], and are summarised briefly here. The studies included treatment‐naïve or treatment‐experienced CHB patients with HBV DNA ≥ 20,000 IU/mL, ALT > 60 U/L in men or > 38 U/L in women, with or without compensated cirrhosis, eGFR_CG_ ≥ 50 mL/min and no evidence of HCC by recent imaging. Full inclusion and exclusion criteria are provided in Supporting Information (Table S1). Patients included in this analysis were enrolled at study sites in five East Asian countries: Hong Kong, Japan, Singapore, South Korea and Taiwan (Table S2). The studies were approved by the institutional review board or independent ethics committees at all participating sites and were performed in accordance with the principles of the Declaration of Helsinki and Good Clinical Practice. All patients provided written informed consent before starting study‐related procedures.

As initially designed, patients were to be randomised 2:1 to receive TAF 25 mg or TDF 300 mg, each given orally once daily (with placebo‐to‐match), for 96 weeks followed by open‐label treatment with TAF 25 mg orally once daily until Week 144 (Year 3). Randomisation was stratified by HBV DNA level and treatment status (naïve vs. experienced). Following the primary endpoint analysis at Week 48, an amendment was introduced to the protocol allowing the extension of double‐blind treatment to Week 144, and the open‐label TAF treatment period was extended to Week 384 (Year 8). Implementation of the protocol amendment varied widely across study sites such that approximately half of the patients had already moved to open‐label TAF at Week 96 (Year 2) while the remainder continued double‐blind treatment for an additional year. As a consequence, for this analysis, patients received either 240 weeks (5 years) of continuous TAF treatment (double‐blind treatment for 2 or 3 years, followed by open‐label treatment through Year 5), 96 weeks (2 years) of double‐blind TDF followed by 3 years of open‐label TAF treatment (TDF → TAF 3 years) or 144 weeks (3 years) of double‐blind TDF followed by 2 years of open‐label TAF treatment (TDF → TAF 2 years).

### 2.2 Study Outcomes

Efficacy and safety were analysed using pooled data from both studies. Antiviral efficacy was assessed by evaluation of the proportion of patients with HBV DNA < 29 IU/mL at Weeks 48, 96, 144 and 240 measured using the manual cobas TaqMan HBV test for use with the High Pure System (baseline to Week 144) or the fully automated cobas AmpliPrep/cobas TaqMan HBV Test v2.0 (Week 144 to Week 240) (Roche Diagnostics, Indianapolis, IN, USA). Correlation of samples, reproducibility, linearity and limit of detection (~10 IU/mL) are acceptable and similar between assays [25]. Normalisation of ALT levels was assessed using both the central laboratory upper limit of normal (ULN; men ≤ 43 U/L and women ≤ 34 U/L) and the 2018 American Association for the Study of Liver Diseases (AASLD) ULN (men ≤ 35 U/L and women ≤ 25 U/L) [3]. Other efficacy endpoints included HBeAg loss and seroconversion in HBeAg‐positive patients, hepatitis B surface antigen (HBsAg) loss and seroconversion and change from baseline in quantitative HBsAg.

Safety outcomes included aggregate treatment‐emergent adverse events (AEs) and laboratory abnormalities, which were assessed separately during the double‐blind and open‐label phases. Bone‐related safety outcomes were assessed by visit over both phases and included percentage changes in hip and spine BMD assessed using dual energy X‐ray absorptiometry (DXA) and two widely accepted [26] serum markers of bone turnover (C‐type collagen sequence [CTX], a sensitive marker of bone resorption and procollagen type 1 N‐terminal [P1NP], a sensitive marker of bone formation). Results from hip and spine BMD were evaluated by an independent central reviewer (Clario, formerly Bioclinica Inc., Philadelphia, PA, USA). Renal‐related safety outcomes were assessed by visit and included changes in eGFR_CG_ and serum creatinine, as well as changes in two sensitive markers of proximal tubular function: urine retinol‐binding protein‐to‐creatinine ratio (RBP/Cr) and urine β_2_‐microglobulin‐to‐creatinine ratio (β_2_M/Cr). Changes in fasting serum lipids (total cholesterol [TC], high‐density lipoprotein [HDL] cholesterol, low‐density lipoprotein [LDL] cholesterol, triglycerides, and total/HDL cholesterol ratio) were also assessed.

### 2.3 Statistical Analysis

Data were analysed using SAS software (SAS Institute Inc., Version 9.4, Cary, NC, USA). Categorical endpoints were summarised by number and percentage of patients meeting the endpoint. Continuous endpoints were summarised using descriptive statistics. For the efficacy endpoints involving proportions, missing data were handled using the missing equals failure (M = F) approach. An additional sensitivity analysis of efficacy was performed using a missing equals excluded (M = E) approach. One centre from East Asia elected not to participate in the protocol amendment; therefore, 25 patients from Study 108 and 44 patients from Study 110 completed participation at Year 3 and are excluded from the study population in this 5‐year analysis. Given the differing times for patients rolling over to open‐label treatment, statistical comparisons between groups for efficacy and safety were not performed.

## 3 Results

Of the 1298 patients randomised and treated in studies 108 and 110, 591 (46%) were enrolled from East Asian study sites: [27] 401/591 (68%) patients received TAF throughout the study, while 84 and 106 patients (total 190/591; 32%) switched from TDF to TAF at Week 96 (TDF → TAF 3 years group) or Week 144 (TDF → TAF 2 years group), respectively. Patient characteristics at baseline were generally well balanced, particularly with respect to levels of HBV DNA and serum ALT. As would be expected in a subanalysis from this region, nearly all patients were either HBV genotype B or C (Table 1).

**TABLE 1:** Baseline demographics and disease characteristics.

	TAF (*n* = 401)	TDF → TAF 3 years (*n* = 84)	TDF → TAF 2 years (*n* = 106)
Mean age, years (SD)	43 (10.8)	45 (11.6)	45 (11.1)
Male, *n* (%)	232 (57.9)	41 (48.8)	67 (63.2)
Mean BMI (SD)	23.8 (3.7)	24.0 (4.0)	24.5 (4.0)
BMI category, kg/m^2^, *n* (%)			
< 18.5 (underweight)	17 (4.2)	1 (1.2)	8 (7.5)
≥ 18.5–25.0 (normal)	250 (62.3)	55 (65.5)	56 (52.8)
> 25.0–30.0 (overweight)	115 (28.7)	22 (26.2)	33 (31.1)
> 30.0 (obese)	19 (4.7)	6 (7.1)	9 (8.5)
HBeAg positive, *n* (%)	284 (70.8)	53 (63.1)	73 (68.9)
HBV DNA, log_10_ IU/mL			
Mean (SD)	7.0 (1.51)	6.9 (1.59)	6.9 (1.51)
≥ 8, *n* (%)	119 (29.7)	23 (27.4)	30 (28.3)
Median ALT, U/L (Q1, Q3)	80 (56, 126)	78 (56, 117)	82 (52, 137)
HBV genotype, *n* (%)			
B	92 (22.9)	30 (35.7)	26 (24.5)
C	308 (76.8)	52 (61.9)	76 (71.7)
Other^a^	1 (0.2)	2 (2.4)	4 (3.8)
Patients with known cirrhosis, *n*/*N* (%)	38/300 (12.7)	12/69 (17.4)	8/78 (10.3)
Mean FibroTest score^b^ (SD)	(*n* = 394)	(*n* = 82)	(*n* = 106)
	0.39 (0.23)	0.36 (0.22)	0.41 (0.25)
FibroTest score ≥ 0.75,^b^ *n*/*N* (%)	40/394 (10.2)	6/82 (7.3)	17/106 (16.0)
Previous nucleos(t)ide use, *n* (%)	100 (24.9)	14 (16.7)	22 (20.8)
Median eGFR_CG_, mL/min	104.4	98.4	99.6
(Q1, Q3)	(87.6, 123.0)	(79.5, 115.2)	(91.2, 115.8)
Osteoporosis by hip BMD *T*‐score,^c^ *n* (%)	9 (2.3)	1 (1.2)	0
Osteoporosis by spine BMD *T*‐score,^c^ *n* (%)	28 (7.0)	12 (14.3)	6 (5.7)
Osteopenia by hip BMD *T*‐score,^d^ *n* (%)	136 (34.0)	35 (41.7)	35 (33.0)
Osteopenia by spine BMD *T*‐score,^d^ *n* (%)	144 (35.9)	33 (39.3)	38 (35.8)
Diabetes mellitus, *n* (%)	27 (6.7)	3 (3.6)	13 (12.3)
Hypertension, *n* (%)	56 (14.0)	14 (16.7)	18 (17.0)
Hyperlipidaemia, *n* (%)	52 (13.0)	12 (14.3)	13 (12.3)
Lipid‐modifying medications, *n* (%)	19 (4.7)	2 (2.4)	4 (3.8)
Cardiovascular disease, *n* (%)	14 (3.5)	1 (1.2)	4 (3.8)
Fasting LDL cholesterol > 190 mg/dL, *n*/*N* (%)	6/397 (1.5)	2/83 (2.4)	4/106 (3.8)
Fasting triglycerides > 750 mg/dL, *n*/*N*	0/397	0/83	0/106

Abbreviations: ALT, alanine aminotransferase; BMD, bone mineral density; BMI, body mass index; eGFR_CG_, estimated glomerular filtration rate by Cockcroft‐Gault; HBeAg, hepatitis B e antigen; HBV, hepatitis B virus; LDL, low‐density lipoprotein; Q, quartile; SD, standard deviation; TAF, tenofovir alafenamide; TDF, tenofovir disoproxil fumarate; ULN, upper limit of normal.

^a^ Seven patients infected with other/unknown.

^b^ Conversion of FibroTest score to histological classification by METAVIR: ≤ 0.27: F0; ≤ 0.48: F1; ≤ 0.58: F2; ≤ 0.74: F3; 0.75–1.00: F4 (consistent with cirrhosis) [28].

^c^ Osteoporosis defined as *T*‐score < −2.5.

^d^ Osteopenia defined as *T*‐score −2.5 to < −1.0.

### 3.1 Efficacy

**FIGURE 1:**
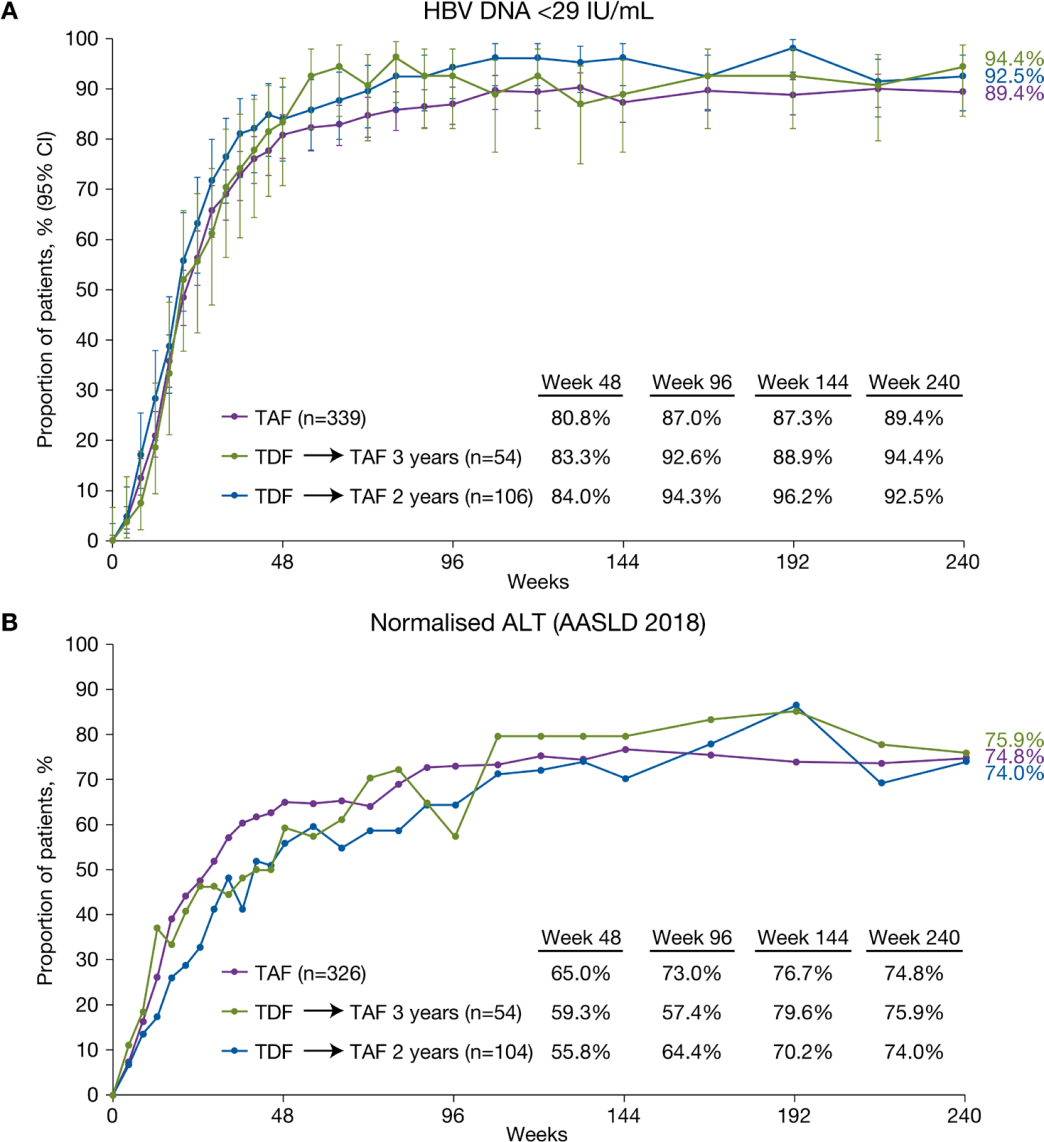
Viral suppression and ALT normalisation. (A) Proportion of patients with HBV DNA < 29 IU/mL by study visit over 5 years. (B) Proportion of patients with ALT >ULN at baseline achieving ALT normalisation by study visit over 5 years using AASLD 2018 [3] (men ≤ 35 U/L and women ≤ 25 U/L) criteria. Missing = failure analysis. AASLD, American Association for the Study of Liver Diseases; ALT, alanine amino transferase; TAF, tenofovir alafenamide; TDF, tenofovir disoproxil fumarate.

Virologic control was achieved early (by Week 48) and was maintained in most patients receiving TAF throughout the 5‐year study period and for those receiving TDF who switched to TAF at Week 96 or 144 (Table 2, Figure 1A). The proportion of patients with HBV DNA < 29 IU/mL at Year 5 was similar among treatment groups: 89% in the TAF group, 94% in the TDF → TAF 3 years group and 92% in the TDF → TAF 2 years group (Table 2, Figure 1A), with HBV DNA levels undetectable in 45%, 59% and 49% of patients, respectively. Normalisation of ALT levels was achieved by 85% in the TAF group, 90% in the TDF → TAF 3 years group and 78% in the TDF → TAF 2 years group when assessed by central laboratory criteria, and in 75%, 76% and 74%, respectively, by AASLD 2018 criteria [3] (Table 2, Figure 1B). Median (quartile 1 [Q1], quartile 3 [Q3]) ALT levels at Year 5 were 20.0 (14.0, 30.0) U/L in the TAF group, 20.0 (13.0, 26.0) U/L in the TDF → TAF 3 years group and 21.5 (15.0, 31.0) U/L in the TDF → TAF 2 years group (Table S3). Median (Q1, Q3) change from baseline in ALT was similar among groups. The M = E virologic and biochemical data are provided in Table S3.

**TABLE 2:** Viral, biochemical and serologic responses at Year 5 (missing = failure analysis).

	TAF (*n* = 401)	TDF → TAF 3 years (*n* = 84)	TDF → TAF 2 years (*n* = 106)
HBV DNA < 29 IU/mL, *n*/*N* (%) [95% CI]	303/339 (89.4)	51/54 (94.4)	98/106 (92.5)
	[85.6, 92.5]	[84.6, 98.8]	[85.7, 96.7]
HBV DNA < 29 IU/mL with TND	153/339 (45.1)	32/54 (59.3)	52/106 (49.1)
HBV DNA ≥ 29 IU/mL, *n*/*N* (%)	36/339 (10.6)	3/54 (5.6)	8/106 (7.5)
HBV DNA 29–< 69 IU/mL	10/339 (2.9)	0	1/106 (0.9)
HBV DNA ≥ 69 IU/mL	7/339 (2.1)	0	1/106 (0.9)
Missing	19/339 (5.6)	3/54 (5.6)	6/106 (5.7)
Normalised ALT^a^ (central laboratory^b^), *n*/*N* (%)	256/302 (84.8)	46/51 (90.2)	76/97 (78.4)
Normalised ALT^a^ (AASLD^c^), *n*/*N* (%)	244/326 (74.8)	41/54 (75.9)	77/104 (74.0)
HBeAg			
Loss, *n*/*N* (%)	86/240 (35.8)	15/35 (42.9)	32/72 (44.4)
Seroconversion, *n*/*N* (%)	61/240 (25.4)	12/35 (34.3)	20/72 (27.8)
HBsAg			
Loss, *n*/*N* (%)	1/339 (0.3)	0/54	1/106 (0.9)^d^
Median change at Week 240, log_10_ IU/mL (Q1, Q3)	*n* = 320–0.26 (−0.77, 0.03)	*n* = 51–0.24 (−0.88, 0.13)	*n* = 100–0.37 (−0.96, −0.09)

Abbreviations: AASLD, American Association for the Study of Liver Diseases; ALT, alanine aminotransferase; CI, confidence interval; HBeAg, hepatitis B e antigen; HBsAg, hepatitis B surface antigen; HBV, hepatitis B virus; Q, quartile; TAF, tenofovir alafenamide; TDF, tenofovir disoproxil fumarate; TND, target not detected; ULN, upper limit of normal.

^a^ ALT normalisation: ≤ ULN in patients with ALT > ULN at baseline.

^b^ Central laboratory ULN: men ≤ 43 U/L; women ≤ 34 U/L (≥ 69 years old, men ≤ 35 U/L; women ≤ 32 U/L).

^c^ 2018 AASLD ULN (Terrault 2018) [3]: men ≤ 35 U/L; women ≤ 25 U/L.

^d^ Patient also achieved seroconversion.

Rates of HBeAg loss and seroconversion increased progressively across groups over 5 years, while overall, few patients (< 1%) achieved HBsAg loss during this period (Table 2). In support of the low response rate for HBsAg loss, quantitative HBsAg levels decreased minimally over 5 years in all groups; median change at Year 5 was −0.26 log_10_ IU/mL in the TAF group, −0.24 log_10_ IU/mL in the TDF → TAF 3 years group and −0.37 log_10_ IU/mL in the TDF → TAF 2 years group (Table 2, Figure S1).

### 3.2 Safety

Overall, TAF treatment or switching from TDF to TAF in CHB patients of East Asian ethnicity was well tolerated over 5 years. Rates of Grade 3 or 4 AEs were low in both the double‐blind (Table 3a) and open‐label (Table 3b) phases, and few AEs were considered by the investigator to be related to the study treatment. Rates of treatment discontinuation due to an AE were low across treatment groups.

**TABLE 3a:** Adverse events during the double‐blind phase (safety analysis set).

	TAF (*n* = 401)	TDF → TAF 3 years (*n* = 84)	TDF → TAF 2 years (*n* = 106)
Adverse events, *n* (%)			
Any AE	326 (81.3)	61 (72.6)	90 (84.9)
Grades 3 and 4 AE	29 (7.2)	5 (6.0)	4 (3.8)
Grades 3 and 4 AE related to study drug	0	0	0
Serious AE	44 (11.0)	9 (10.7)	13 (12.3)
Serious AE related to study drug	0	0	0
Discontinuation due to AE^a^	5 (1.2)	0	0
Potential cardiovascular events	6 (1.5)	0	0
Death	0	0	0
Grades 3 and 4 laboratory abnormalities in ≥ 2% of patients in any group, *n*/*N* (%)			
Any grades 3 and 4	148/399 (37.1)	26/84 (31.0)	35/106 (33.0)
Haemoglobin < 9.0 g/dL	1/399 (0.3)	2/84 (2.4)	3/106 (2.8)
Platelets < 50 Gi/L	1/399 (0.3)	2/84 (2.4)	1/106 (0.9)
Alanine aminotransferase > 5 × ULN	36/399 (9.0)	5/84 (6.0)	11/106 (10.4)
Aspartate aminotransferase > 5 × ULN	12/399 (3.0)	2/84 (2.4)	5/106 (4.7)
Amylase > 2 × ULN	8/399 (2.0)	2/84 (2.4)	4/106 (3.8)
Creatine kinase ≥ 10 × ULN	12/399 (3.0)	2/84 (2.4)	2/106 (1.9)
Fasting LDL cholesterol > 190 mg/dL	34/397 (8.6)	2/84 (2.4)	0/106
Urine glucose ≥ 4 mmol/L	19/399 (4.8)	0	4/106 (3.8)
Urine erythrocytes > 75 RBC/HPF	42/383 (11.0)	6/79 (7.6)	13/104 (12.5)

Abbreviations: AE, adverse event; HCC, hepatocellular carcinoma; LDL, low‐density lipoprotein; RBC/HPF, red blood cells per high‐power field; TAF, tenofovir alafenamide; TDF, tenofovir disoproxil fumarate; ULN, upper limit of normal.

^a^ AEs leading to study discontinuation: basilar artery occlusion (*n* = 1), symptoms of dyspepsia, nausea, and vomiting (*n* = 1), pancreatic carcinoma (*n* = 2) and HCC (*n* = 1).

**TABLE 3b:** Adverse events during the open‐label phase (open‐label safety analysis set).

	TAF (*n* = 373)	TDF → TAF 3 years (*n* = 84)	TDF → TAF 2 years (*n* = 106)
Adverse events, *n* (%)			
Any AE	225 (60.3)	47 (56.0)	61 (57.5)
Grades 3 and 4 AE	20 (5.4)	6 (7.1)	4 (3.8)
Grades 3 and 4 AE related to study drug^a^	1 (0.3)	0	0
Serious AE	42 (11.3)	10 (11.9)	10 (9.4)
Serious AE related to study drug^b^	2 (0.5)	0	0
Discontinuation due to AE^c^	2 (0.5)	0	1 (0.9)
Potential cardiovascular events	5 (1.3)	3 (3.6)	1 (0.9)
Death	0	0	0
Grades 3 and 4 laboratory abnormalities in ≥ 2% of patients in any group, *n*/*N* (%)			
Any grades 3 and 4	54/370 (14.6)	13/83 (15.7)	15/106 (14.2)
Amylase > 2 × ULN	3/370 (0.8)	3/83 (3.6)	2/106 (1.9)
Fasting glucose > 250 mg/dL	3/368 (0.8)	2/81 (2.5)	3/106 (2.8)
Fasting LDL cholesterol > 190 mg/dL	15/368 (4.1)	3/81 (3.7)	5/106 (4.7)
Fasting triglycerides > 750 mg/dL	0	2/81 (2.5)	0
Urine erythrocytes > 75 RBC/HPF	15/275 (5.5)	1/71 (1.4)	2/74 (2.7)
Urine glucose ≥ 4 mmol/L	10/370 (2.7)	1/83 (1.2)	2/106 (1.9)

Abbreviations: AE, adverse event; LDL, low‐density lipoprotein; RBC/HPF, red blood cells per high‐power field; TAF, tenofovir alafenamide; TDF, tenofovir disoproxil fumarate; ULN, upper limit of normal.

^a^ Grades 3 and 4 AE considered related to TAF: renal neoplasm.

^b^ Serious AEs considered related to TAF: renal neoplasm (*n* = 1) and osteonecrosis (*n* = 1).

^c^ AEs leading to study discontinuation: osteonecrosis and pancreatic carcinoma (TAF) and pemphigoid (TDF → TAF 2 years).

**FIGURE 2:**
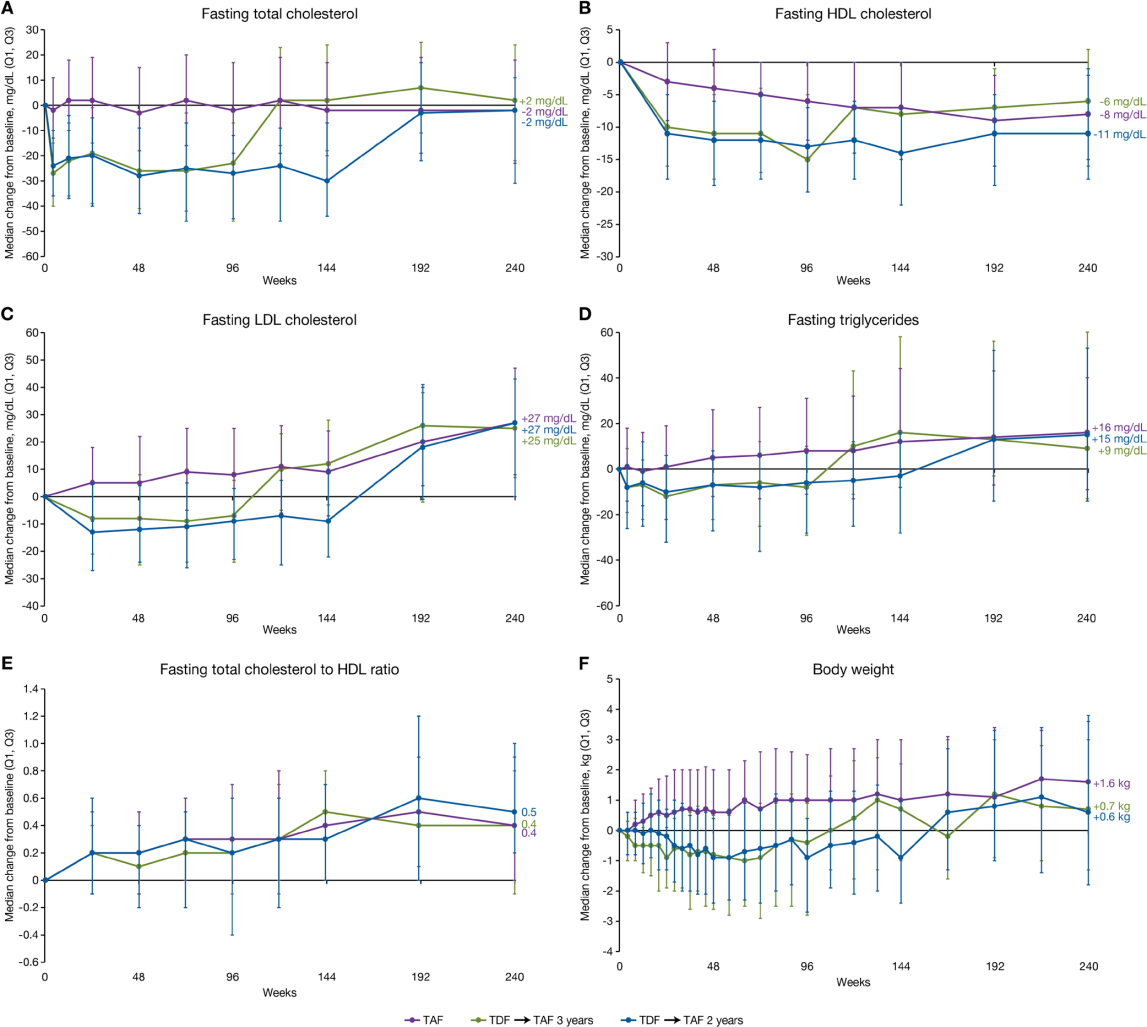
Changes in fasting lipids and body weight. Median changes in (A) fasting total cholesterol, (B) fasting HDL cholesterol, (C) fasting LDL cholesterol, (D) fasting triglycerides, (E) fasting total cholesterol/HDL cholesterol ratio and (F) body weight by study week over 5 years. HDL, high‐density lipoprotein; LDL, low‐density lipoprotein; TAF, tenofovir alafenamide; TDF, tenofovir disoproxil fumarate.

During the double‐blind phase of the study, levels of all lipid parameters decreased in patients randomised to TDF (Figure 2A−D). Over the double‐blind phase in those randomised to TAF, median increases in TC (Figure 2A), LDL cholesterol (Figure 2C) and triglycerides (Figure 2D) were observed, which remained stable through Year 5. In patients switched from TDF to TAF, median increases in all lipid parameters were observed, reaching levels comparable to those observed in patients initially randomised to TAF, with these levels tending to remain stable over time. The fasting total/HDL cholesterol ratio was similar between groups throughout the study (Figure 2E). During the double‐blind and open‐label phases of the study, the rates of Grade 3 fasting cholesterol were < 2% in all groups. The incidence of Grade 3 fasting LDL cholesterol is presented in Tables 3a and 3b. No Grade 4 abnormalities in fasting cholesterol or LDL cholesterol were reported. Overall, the rate of potential cardiovascular events was low in both the double‐blind and open‐label phases (Tables 3a and 3b, Table S4). All participants with cardiovascular events had risk factors such as advanced age and/or preexisting diabetes or hypertension or hyperlipidaemia.

The percentage of patients initiating lipid‐modifying medication during the double‐blind phase was 2.6% in the TAF group, 1.2% in the TDF → TAF 3 years group, and 1.0% in the TDF → TAF 2 years group. During the open‐label phase, lipid‐modifying medication was initiated by 4.3% of patients in the TAF group, 3.7% in the TDF → TAF 3 years group, and 4.0% in the TDF → TAF 2 years group. Two patients in the TAF group who were taking lipid‐modifying medication at baseline were diagnosed with HCC. Median changes in body weight from baseline to Year 5 were +1.6 kg in the TAF group, +0.7 kg in the TDF → TAF 3 years group, and +0.6 kg in the TDF → TAF 2 years group (Figure 2F).

**FIGURE 3:**
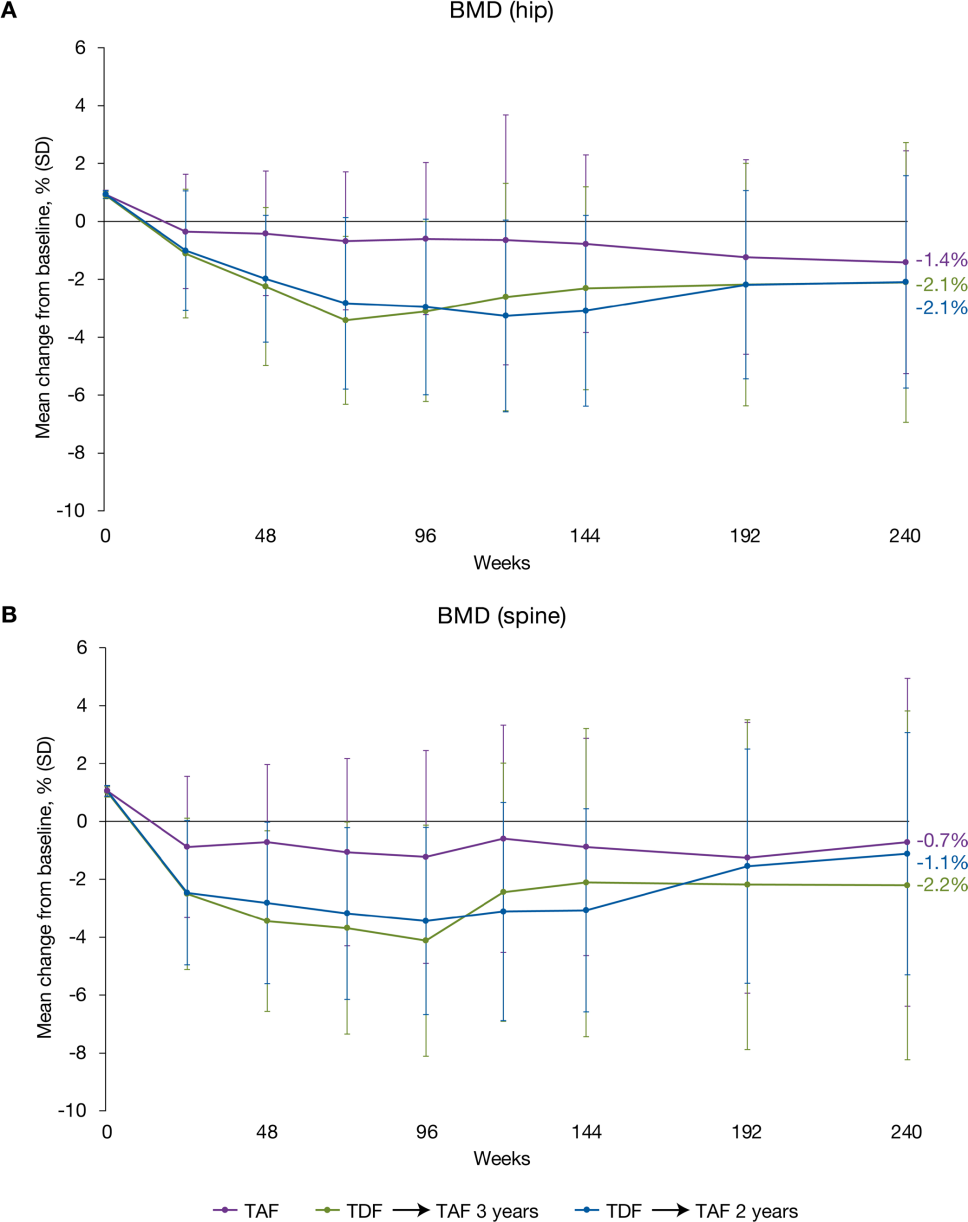
Changes in BMD. Mean percentage change in hip (A) and (B) spine BMD from baseline by study visit over 5 years of treatment. BMD, bone mineral density; SD, standard deviation; TAF, tenofovir alafenamide; TDF, tenofovir disoproxil fumarate.

In patients receiving TAF throughout the study, mean changes from baseline in hip and spine BMD at Year 5 were small (Figure 3). In patients initially randomised to TDF, greater decreases in BMD were observed during the double‐blind phase, with improvement noted after switching to TAF (Figure 3). This was reflected in changes in markers of bone resorption (CTX, Figure S2A) and bone formation (P1NP, Figure S2B). In patients receiving TDF during the double‐blind portion of the study, levels of CTX and P1NP increased initially and decreased after switching to TAF at Week 96 or 144. At Year 5, median changes from baseline were similar between groups for both biomarkers.

**FIGURE 4:**
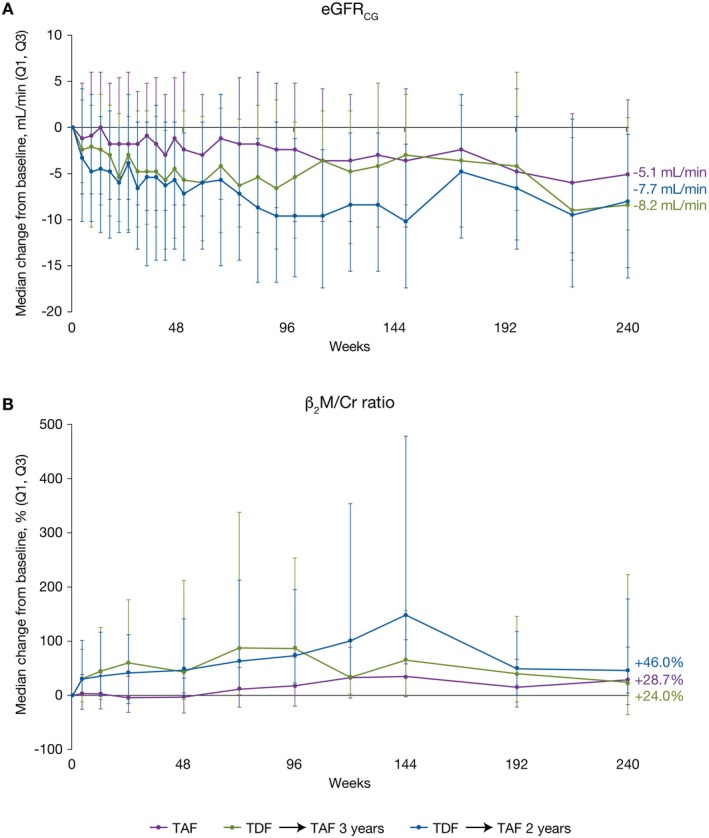
Changes in eGFR_CG_ and β_2_M/Cr. Median change from baseline in eGFR_CG_ (A) and serum β_2_M/Cr (B) by study visit over 5 years. eGFR_CG_, estimated glomerular filtration rate by Cockcroft‐Gault; TAF, tenofovir alafenamide; TDF, tenofovir disoproxil fumarate; β_2_M/Cr, urine β2‐microglobulin‐to‐creatinine.

In terms of renal safety, patients randomised to TAF had minimal changes in eGFR_CG_ during the double‐blind phase, with levels remaining relatively stable during the open‐label phase (Figure 4A). The greater decreases observed in the double‐blind phase in patients randomised to TDF largely reversed on switching to TAF. All groups showed a marginal increase in serum creatinine levels from baseline to Year 5 (Figure S3). For the β_2_M/Cr (Figure 4B) and RBP/Cr (Figure S4) ratios, patients receiving TDF during the double‐blind phase of the study showed greater increases compared with patients receiving TAF. These changes improved upon switching to TAF.

Twelve cases of HCC occurred during the 5‐year study period (12/591 [2.0%]): eight cases emerged during double‐blind treatment and four cases occurred during the open‐label phase. Seven HCC cases occurred in the TAF group (7/401 [1.7%]; five in the double‐blind phase and two in the open‐label phase) and five HCC cases occurred in the TDF → TAF groups (5/190 [2.6%]; three in the double‐blind phase and two in the open‐label phase). No cases of HCC were considered related to study treatment. One patient discontinued treatment due to HCC.

## 4 Discussion

Our results, derived from a subset of East Asian patients with HBeAg‐positive or HBeAg‐negative CHB enrolled in two multinational Phase 3 studies, are comparable to those previously published for the global population [16], as well as for a separate cohort of CHB patients enrolled in these two studies in China [17]. High proportions of patients in all three treatment groups achieved and maintained viral suppression over 5 years. Importantly, during double‐blind treatment at Weeks 48 and 96 in the overall study population, rates of viral suppression were consistent across pre‐specified subgroups, including patients of Asian or non‐Asian ethnicity, lending further support to the applicability of the present findings [14, 15, 16, 27]. Results from our analysis are also consistent with those previously reported for Asian patients with CHB treated with TDF through 5 years [29].

High rates of ALT normalisation were seen across all treatment groups at Year 5 and were comparable to the global population results [16]. Rates of ALT normalisation were higher with TAF during the double‐blind phase and increased in TDF‐treated patients after switching to TAF, a trend that has been reported in multiple studies, controlled trials and in routine clinical practice [19, 22]. In a Phase 3 study of virally suppressed CHB patients on long‐term TDF treatment, switching to TAF resulted in higher rates of ALT normalisation at Week 48 compared with continued TDF treatment (50% vs. 26%, respectively; *p* = 0.014) [19]. Similar findings were reported from a retrospective analysis conducted in Hong Kong that followed consecutive CHB patients switched from TDF to TAF for at least 12 months [30]. In that study, median ALT was also lower at Month 12 than at baseline (21.0 U/L vs. 25.0 U/L, respectively, *p* < 0.001) and the ALT normalisation rate was significantly higher (90% vs. 84%, respectively, *p* = 0.037). This finding is relevant given that analyses of real‐world cohorts have shown that antiviral treatment leading to early ALT normalisation within 12 months is associated with a lower risk of hepatic events (including HCC) in patients with CHB [31, 32, 33]. Normal ALT levels were also associated with lower HCC incidence in the PAGE‐B natural history cohort study [34].

Rates of HBsAg loss reported in this analysis were numerically lower than those reported in the overall cohort [14, 15, 16]. This could be associated with a high proportion of patients infected with genotypes B and C in the present analysis. A previous analysis of long‐term TDF results in HBeAg‐positive CHB patients suggested a greater likelihood of achieving HBsAg loss in patients infected with genotypes A or D [35]. Our results are consistent with those of numerous other studies showing that antiviral treatment rarely leads to HBsAg loss in HBV monoinfection, including in Asian patients [35, 36, 37, 38, 39, 40, 41, 42].

Overall, we found study treatment to be well tolerated across treatment groups through 5 years, with low rates of serious AEs and discontinuations due to AEs. Although weight gain was seen in all groups, median weight gain was less than the reported approximate 1 kg average per year in the NHANES and CARDIA cohorts [43, 44]. Although the study was not designed to assess cardiovascular risk factors or outcomes, in this analysis of East Asian patients, increases in TC, HDL cholesterol, LDL cholesterol and triglycerides were observed upon switching from TDF to TAF. These findings align with observations from the global 5‐year cohort and routine clinical practice [14, 15, 29, 45, 46, 47], are consistent with the removal of TDF's lipid‐lowering effect [45, 46, 48, 49, 50], and are in line with previous observations that TAF may have a ‘lipid neutral’ effect [27]. This concept is further supported by studies in patients switched from ETV to TAF, in whom increases in lipid levels were not observed [22]. Analysis of the fasting total/HDL cholesterol ratio, which is considered a more accurate predictor of cardiovascular disease risk than individual lipid parameters [51], showed similar changes over time for all groups with no overall impact on cardiovascular risk [52, 53].

During the double‐blind comparison of TAF vs. TDF in the overall study population, statistically superior bone and renal outcomes were seen at Weeks 48 and 96. Importantly, these outcomes in the present analysis of East Asian patients were consistent with the overall study population at Weeks 48 and 96, and also at Year 5 [14, 15, 16, 27]. Hip and spine BMD remained relatively stable over 5 years in patients treated with TAF, and early declines observed in patients treated with TDF steadily improved after switching to TAF. Results from meta‐analyses have shown that TDF treatment is associated with decreased renal function in patients with HIV or HBV [54, 55]. In the present analysis, patients receiving TDF showed greater decreases in eGFR_CG_ compared with those receiving TAF during the double‐blind portion of the studies. After switching to TAF at Week 96 or 144, eGFR_CG_ levels increased. These results are consistent with those reported for the overall study populations at Weeks 48 and 96 and at Year 5 [14, 15, 16, 27]. These changes in renal parameters are in line with reported changes in markers of proximal renal tubule damage [9, 10].

Variation between centres with respect to when the protocol amendment extending the double‐blind phase of treatment was implemented divided the TDF‐treated group into two subgroups and precluded statistical comparisons between groups. Despite this limitation, the 5‐year efficacy and safety data for TAF treatment were largely consistent across treatment groups.

With CHB patients ageing, and Asian patients at particular risk of osteoporosis and renal dysfunction, the occurrence of comorbidities such as chronic kidney disease, osteoporosis, and fractures is of concern [56, 57, 58]. Decreases in BMD or renal function associated with TDF treatment are of clinical concern in patients with CHB due to the need for long‐term NA treatment [4]. After 5 years of treatment, virologic suppression, ALT normalisation and HBeAg loss remained high in East Asian patients treated with TAF or switched from TDF to TAF. Both treatments were well tolerated, with improved renal and bone safety reported in patients switching from TDF to TAF. The findings from the present analysis serve to reassure clinicians treating predominantly Asian populations that the global study results apply to their patients.

## Author Contributions

**Grace Lai‐Hung Wong:** investigation, writing – review and editing. **Edward Gane:** investigation, writing – review and editing. **Calvin Q. Pan:** investigation, writing – review and editing. **Scott Fung:** investigation, writing – review and editing. **Mang M. Ma:** investigation, writing – review and editing. **Namiki Izumi:** investigation, writing – review and editing. **Shalimar:** investigation, writing – review and editing. **Seng Gee Lim:** investigation, writing – review and editing. **Wan‐Long Chuang:** investigation, writing – review and editing. **Rajiv Mehta:** investigation, writing – review and editing. **Young‐Suk Lim:** investigation, writing – review and editing. **Leland J. Yee:** conceptualization, writing – review and editing, methodology. **John F. Flaherty:** conceptualization, writing – review and editing, methodology. **Frida Abramov:** writing – review and editing. **Hongyuan Wang:** formal analysis, data curation, writing – review and editing. **Maria Buti:** conceptualization, investigation, writing – review and editing, methodology.

## Disclosure

Authorship statement: Grace Lai‐Hung Wong and Maria Buti are the guarantors of the article.

## Conflicts of Interest

G.L.‐H.W. reports advisory committee membership for AstraZeneca, Gilead Sciences, GSK, Janssen and Virion Therapeutics; speaker fees for Abbott, AbbVie, Bristol Myers Squibb, Echosens, Ferring, Gilead Sciences, GSK, Janssen and Roche; and research grants from Gilead Sciences. E.G. reports speaker/consultancy fees and advisory board membership for AbbVie, Aligos, Arbutus, Arrowhead, Assembly, Epigenic, Gilead Sciences, GSK, Intellia, Janssen, Merck, Novartis, Precision Bio, Roche and Vir Bio. C.Q.P. reports receipt of institutional research grants from Gilead Sciences and Wuxi‐Hisky. S.F. reports speaker/consultancy fees and advisory board membership for AbbVie, Gilead Sciences, GSK and Novo‐Nordisk. N.I. reports speaker/consultancy fees and advisory board membership for Gilead Sciences. S.G.L. reports advisory board membership for Abbott, Aligos Therapeutics, Arbutus, Assembly, AusperBio, Gilead Sciences, Grifols, GSK, Roche and Sysmex; speaker fees for Abbott, Gilead Sciences, GSK, Roche and Sysmex; and educational/research funding for Abbott, Gilead Sciences, Roche and Sysmex. W.‐L.C. reports advisory board membership for Bristol Myers Squibb, Gilead Sciences, PharmaEssentia Vaccitech; and speaker fees for AbbVie, Bristol Myers Squibb, Gilead Sciences and Roche. Y.‐S.L. reports speaker/consultancy fees and advisory board membership for Gilead Sciences. M.B. reports speaker/consultancy fees and advisory board membership for AbbVie, Gilead Sciences, GSK and Janssen. L.J.Y., J.F.F., F.A. and H.W. are former/current employees of Gilead Sciences and/or hold stocks and shares in Gilead Sciences. Dr. S., R.M. and M.M.M. report no conflicts of interest.

## Supporting information


**Data S1:** apt70327‐sup‐0001‐Supinfo.docx.

## Data Availability

Gilead Sciences shares anonymized individual patient data upon request or as required by law or regulation with qualified external researchers based on submitted curriculum vitae and reflecting non‐conflict of interest. The request proposal must also include a statistician. Approval of such requests is at Gilead Science's discretion and is dependent on the nature of the request, the merit of the research proposed, the availability of the data, and the intended use of the data. Data requests should be sent to datasharing@gilead.com.
