# Supplementary material for: Efficacy and Safety of Tenofovir Alafenamide (TAF) and Tenofovir Disoproxil Fumarate (TDF) Followed by TAF in Chronic Hepatitis B Patients of East Asian Ethnicity Following 5 Years of Treatment
Source: Aliment Pharmacol Ther. 2025 Aug 12;63(1):132–44. doi: 10.1111/apt.70327 (PMC12690228; doi:10.1111/apt.70327)
Supplement: Supplementary file 1 — Data S1: apt70327‐sup‐0001‐Supinfo.docx. [file APT-63-132-s001.docx]

**Supplementary Table S1. Inclusion and exclusion criteria for Study 108 (HBeAg-negative) and Study 110 (HBeAg-positive)**

| **Inclusion criteria** | **Exclusion criteria** |
| --- | --- |
| - Age ≥18 years - HBV DNA ≥20,000 IU/mL - Serum ALT > 60 U/L in men or >38 U/L in women - Creatine clearance of ≥50 mL/min^†^ | - Serum ALT or AST >10 times ULN - Previous interferon use within 6 months of baseline - Platelets ≤50,000 cells/μL - Haemoglobin <10 g/dL - Albumin <3 g/dL - Direct bilirubin >2.5 times ULN - Evidence of decompensation (ie, clinical ascites, encephalopathy, or variceal haemorrhage) - Hepatocellular carcinoma - HCV, HDV, or HIV coinfection |

^†^By Cockcroft‒Gault method. ALT, alanine aminotransferase; AST, aspartate aminotransferase; HBeAg, hepatitis B e antigen; HCV, hepatitis C virus; HDV, hepatitis delta virus; HIV, human immunodeficiency virus; ULN, upper limit of normal.

**Supplementary Table S2a. Patient enrolment in Study 108 in East Asia by country and investigator**

| **Patients enrolled, n (%)** | **Initial randomised therapy** | | **Total**  **N=178** |
| --- | --- | --- | --- |
|  | **TAF 25 mg**  **(n=114)** | **TDF 300 mg^†^**  **(n=64)** |  |
| **Hong Kong**  M-F Yuen  HL-Y Chan  AJ Hui  O Tsang | **41 (36.0)** 12 17 9 3 | **28 (43.8)** 13 7 7 1 | **69 (38.8)** 25 24 16 4 |
| **Japan**  M Saito  N Izumi  H Yatsuhashi  Y Asahina  N Furusho  T Ide  H Nomura  N Sakamoto  T Inokuma  Y Osaki  T Takehara | **21 (18.4)** 5 2 2 2 2 2 2 1 1 1 1 | **6 (9.4)** 1 3 1 0 0 0 0 1 0 0 0 | **27 (15.2)** 6 5 3 2 2 2 2 2 1 1 1 |
| **South Korea**  Y-S Lim  SH Ahn  S-H Bae  HJ Kim  KS Lee  WY Tak  BK Jang  KT Yoon  SW Paik  KS Byun | **30 (26.3)** 7 1 5 3 4 3 2 2 2 1 | **15 (23.4)** 4 5 1 2 0 1 1 1 0 0 | **45 (25.3)** 11 6 6 5 4 4 3 3 2 1 |
| **Taiwan**  W-L Chuang  T-T Chang  J-H Kao  C-T Hu  S-S Yang | **22 (19.3)** 11 5 4 1 1 | **15 (23.4)** 5 3 2 3 2 | **37 (20.8)** 16 8 6 4 3 |

^†^Four patients initially randomised to TDF did not enter the open-label phase of the study and were not included in the current analysis. TAF, tenofovir alafenamide; TDF, tenofovir disoproxil fumarate.

**Supplementary Table S2b. Patient enrolment in Study 110 in East Asia by country and investigator**

| **Patients enrolled, n (%)** | **Initial randomised therapy** | | **Total**  **N=432** |
| --- | --- | --- | --- |
|  | **TAF 25 mg**  **(n=287)** | **TDF 300 mg^†^**  **(n=145)** |  |
| **Hong Kong**  M-F Yuen  HL-Y Chan  AJ Hui  O Tsang  M Li Kin Kong | **71 (24.7)** 25 21 12 11 2 | **50 (34.5)** 19 13 8 7 3 | **121 (28.0)** 44 34 20 18 5 |
| **Japan**  M Saito  H Yatsuhashi  N Izumi  N Furusyo  T Ide  F Ikeda  T Inokuma  Y Itoh  M Nakamuta  Y Osaki  K Takaguchi  N Kawada  H Nomura  Y Asahina  M Omata  N Sakamoto | **35 (12.2)** 4 5 3 3 0 2 2 3 2 2 3 2 2 0 1 1 | **11 (7.6)** 2 0 1 0 3 1 1 0 1 1 0 0 0 1 0 0 | **46 (10.6)** 6 5 4 3 3 3 3 3 3 3 3 2 2 1 1 1 |
| **Singapore**  WC Chow  S-G Lim  EK Teo | **7 (2.4)** 4 2 1 | **2 (1.4)** 0 2 0 | **9 (2.1)** 4 4 1 |
| **South Korea**  HJ Kim  Y-S Lim  KT Yoon  WY Tak  J-S Hwang  SH Ahn  KS Byun  SW Paik  S-H Jeong  YJ Kim  SY Kwon  S-H Bae  J Heo  HS Kim  JS Lee  KS Lee  B Han  SK Baik  SW Cho  W Kim  T Lee  N Park | **120 (41.8)** 15 15 11 8 8 6 4 7 7 3 6 4 4 5 4 3 5 1 1 1 1 1 | **53 (36.6)** 7 7 6 5 2 3 5 1 0 4 1 2 2 1 2 3 0 1 1 0 0 0 | **173 (40.0)** 22 22 17 13 10 9 9 8 7 7 7 6 6 6 6 6 5 2 2 1 1 1 |
| **Taiwan**  W-L Chuang  C-Y Chen  T-T Chang  J-H Kao  C-Y Peng  S-S Yang  T-H Lee  C-T Hu | **54 (18.8)** 16 18 3 4 4 5 3 1 | **29 (20.0)** 11 7 5 2 1 0 1 2 | **83 (19.2)** 27 25 8 6 5 5 4 3 |

^†^15 patients initially randomised to TDF did not enter the open-label phase of the study and were not included in the current analysis. TAF, tenofovir alafenamide; TDF, tenofovir disoproxil fumarate.

**Supplementary Table S3. Viral, biochemical, and serologic responses at Year 5 (missing = excluded analysis)**

|  | | **TAF**  **(n=401)** | **TDF→TAF  3 years**  **(n=84)** | **TDF→TAF  2 years**  **(n=106)** |
| --- | --- | --- | --- | --- |
| HBV DNA <29 IU/mL, n/N (%)  [95% CI] | | 303/320 (94.7)  [91.6, 96.9] | 51/51 (100)  [93.0, 100] | 98/100 (98.0)  [93.0, 99.8] |
| HBV DNA <29 IU/mL with TND | | 153/320 (47.8) | 32/51 (62.7) | 52/100 (52.0) |
| HBV DNA ≥29 IU/mL | | 17/320 (5.3) | 0/51 | 2/100 (2.0) |
| HBV DNA 29–<69 IU/mL | | 10/320 (3.1) | 0/51 | 1/100 (1.0) |
| HBV DNA ≥69 IU/mL | | 7/320 (2.2) | 0/51 | 1/100 (1.0) |
| Median ALT, U/L (Q1, Q3) | | (n=319)  20.0  (14.0, 30.0) | (n=51)  20.0  (13.0, 26.0) | (n=100)  21.5  (15.0, 31.0) |
| Normal ALT^†^ (central laboratory^‡^), n/N (%) | | 284/319 (89.0) | 49/51 (96.1) | 85/100 (85.0) |
| Normalised ALT^†^ (central laboratory^‡^), n/N (%) | | 256/287 (89.2) | 46/48 (95.8) | 76/91 (83.5) |
| Normal ALT^†^ (AASLD^§^), n/N (%) | | 254/319 (79.6) | 41/51 (80.4) | 78/100 (78.0) |
| Normalised ALT^†^ (AASLD^§^),  n/N (%) | | 244/309 (79.0) | 41/51 (80.4) | 77/98 (78.6) |
| HBeAg | Loss, n/N (%) | 86/223 (38.6) | 15/32 (46.9) | 32/68 (47.1) |
|  | Seroconversion, n/N (%) | 61/223 (27.4) | 12/32 (37.5) | 20/68 (29.4) |
| HBsAg | Loss, n/N (%) | 1/319 (0.3) | 0/51 | 1/100 (1.0) |
|  | Seroconversion, n/N (%) | 0/319 | 0/51 | 1/100 (1.0) |

^†^Normal ALT is all patients with levels ≤ULN at the Week 240 timepoint; normalised ALT is patients with ALT >ULN at baseline with levels ≤ULN at the Week 240 timepoint. Central laboratory ULN: men ≤43 U/L; women ≤34 U/L (≥69 years old, men ≤35 U/L; women ≤32 U/L); ^‡^ALT normalisation: ≤ULN at Year 5 in patients with ALT >ULN at baseline; ^§^2018 AASLD ULN^3^: men ≤35 U/L; women ≤25 U/L.

AASLD, American Association for the Study of Liver Diseases; ALT, alanine aminotransferase; CI, confidence interval; HBeAg, hepatitis e surface antigen; HBsAg, hepatitis B surface antigen; HBV, hepatitis B virus; Q, quartile; TAF, tenofovir alafenamide; TDF, tenofovir disoproxil fumarate; TND, target not detected; ULN, upper limit of normal.

**Supplementary Table S4. Potential cardiovascular adverse events**

1. **Double-blind phase (safety analysis set)**

|  | **TAF**  **(n=401)** | **TDF→TAF  3 years**  **(n=84)** | **TDF→TAF  2 years**  **(n=106)** |
| --- | --- | --- | --- |
| Potential cardiovascular events, n (%)  Angina pectoris  Basilar artery occlusion  Increased blood CPK  Carotid arteriosclerosis  Transient ischaemic attack | 6 (1.5)  2 (0.5)  1 (0.2)  1 (0.2)  1 (0.2)  1 (0.2) | 0 | 0 |

1. **Open-label phase (open-label safety analysis set)**

|  | **TAF**  **(n=373)** | **TDF→TAF  3 years**  **(n=84)** | **TDF→TAF  2 years**  **(n=106)** |
| --- | --- | --- | --- |
| Potential cardiovascular events, n (%)  Angina pectoris  Increased blood CPK  Cerebral infarction  Cerebrovascular accident  Coronary artery disease  Coronary artery stenosis  Myocardial ischaemia  Transient ischaemic attack  Vertebrobasilar insufficiency | 5 (1.3)  0  2 (0.5)  0  0  0  1 (0.3)  0  1 (0.3)  1 (0.3) | 3 (3.6)  1 (1.2)  0  1 (1.2)  0  1 (1.2)  0  0  0  0 | 1 (0.9)  0  0  0  1 (0.9)  1 (0.9)  0  0  0  0 |

CPK, creatinine phosphokinase; TAF, tenofovir alafenamide; TDF, tenofovir disoproxil fumarate.

**Supplementary figures**

**
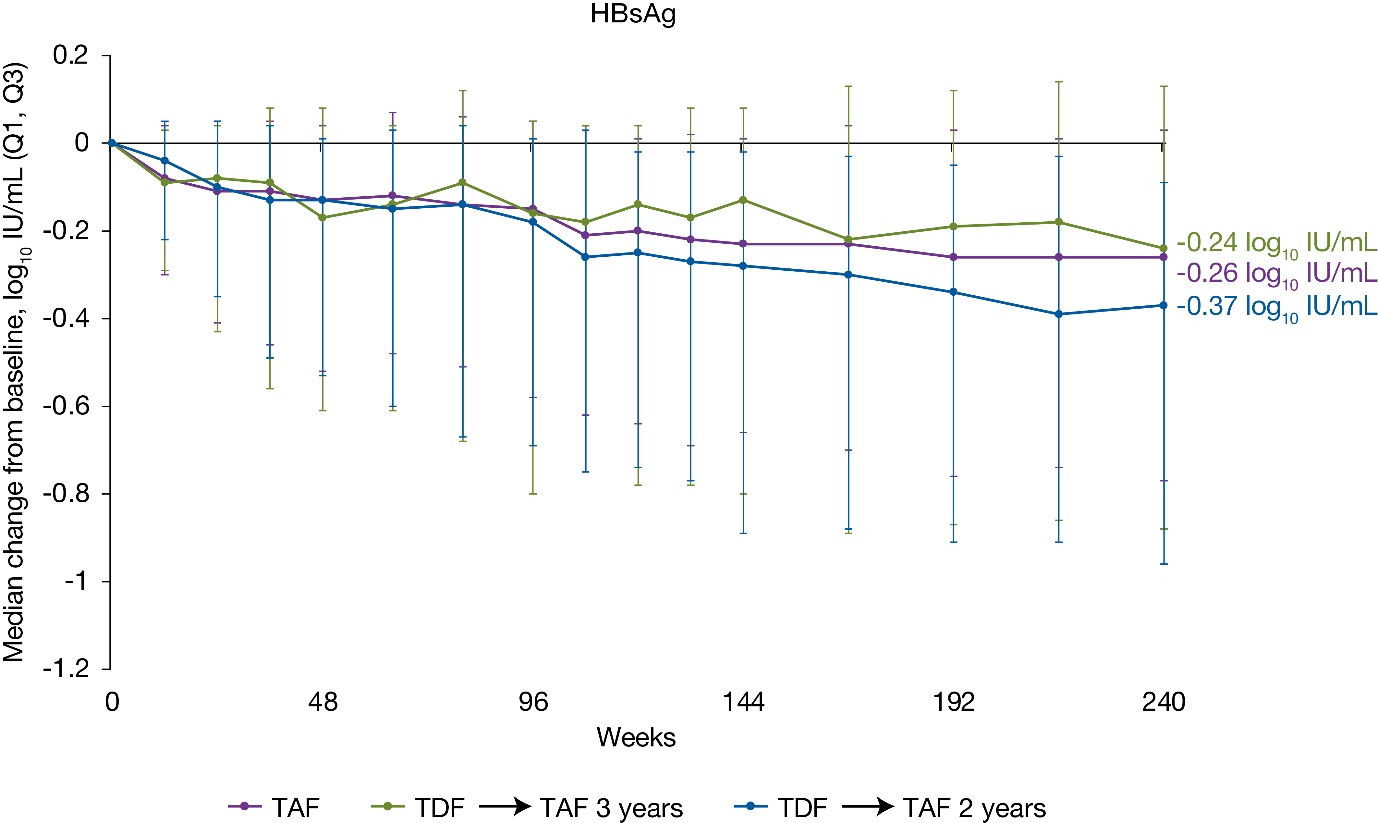
**

**Supplementary Figure S1. Change in HBsAg.** Median change in HBsAg from baseline by study visit over 5 years. HBsAg, hepatitis B surface antigen; Q, quartile; TAF, tenofovir alafenamide; TDF, tenofovir disoproxil fumarate.

**
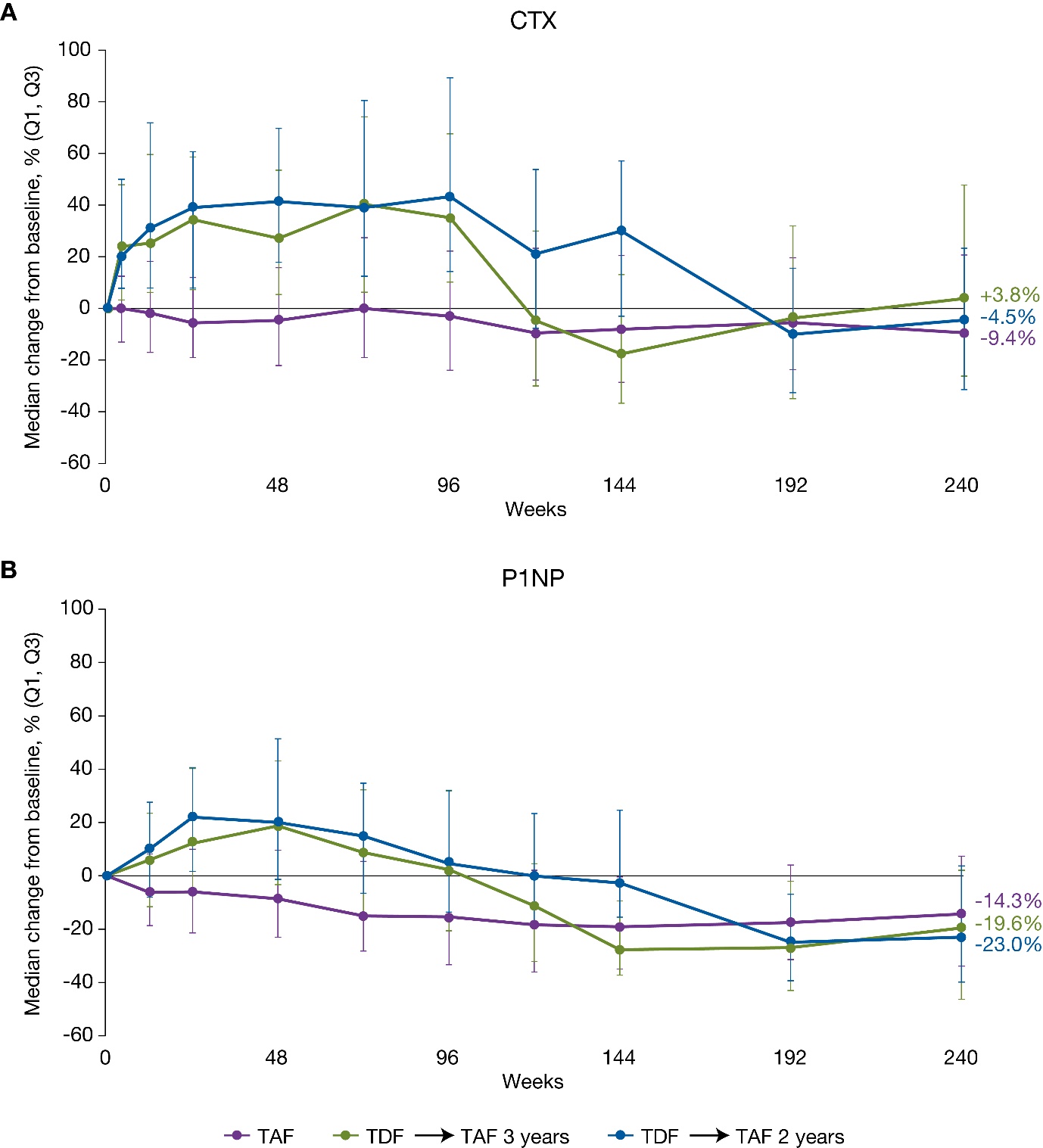
**

**Supplementary Figure S2. Changes in serum bone turnover biomarkers.** Median percentage changes in CTX (A) and P1NP (B) from baseline by study visit over 5 years. CTX, C-type collagen sequence; P1NP, procollagen type 1 N-terminal HBsAg; Q, quartile; TAF, tenofovir alafenamide; TDF, tenofovir disoproxil fumarate.

**
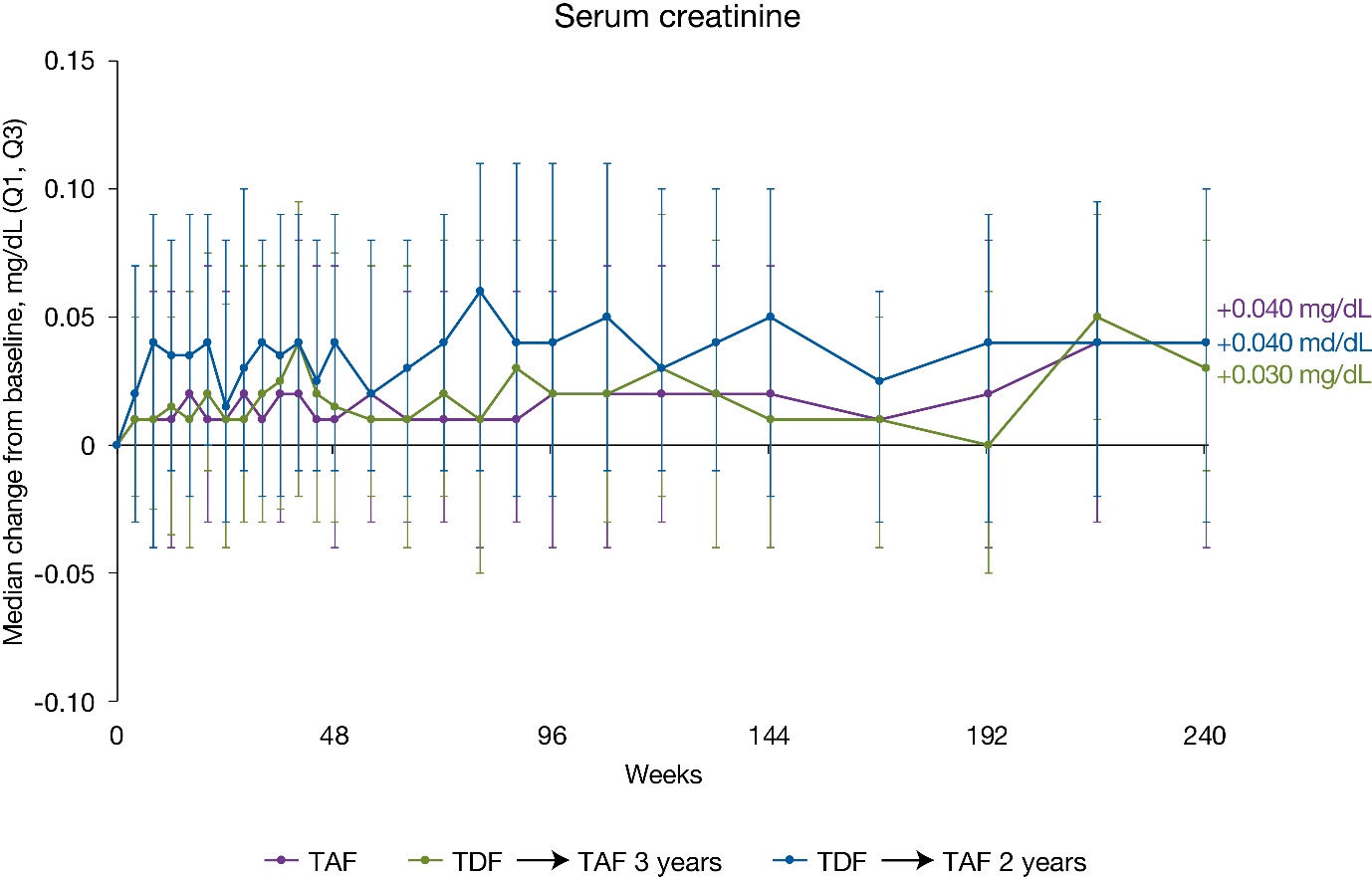
**

**Supplementary Figure S3. Changes in serum creatinine.** Median change from baseline in serum creatinine by study visit over 5 years. Q, quartile; TAF, tenofovir alafenamide; TDF, tenofovir disoproxil fumarate.

**
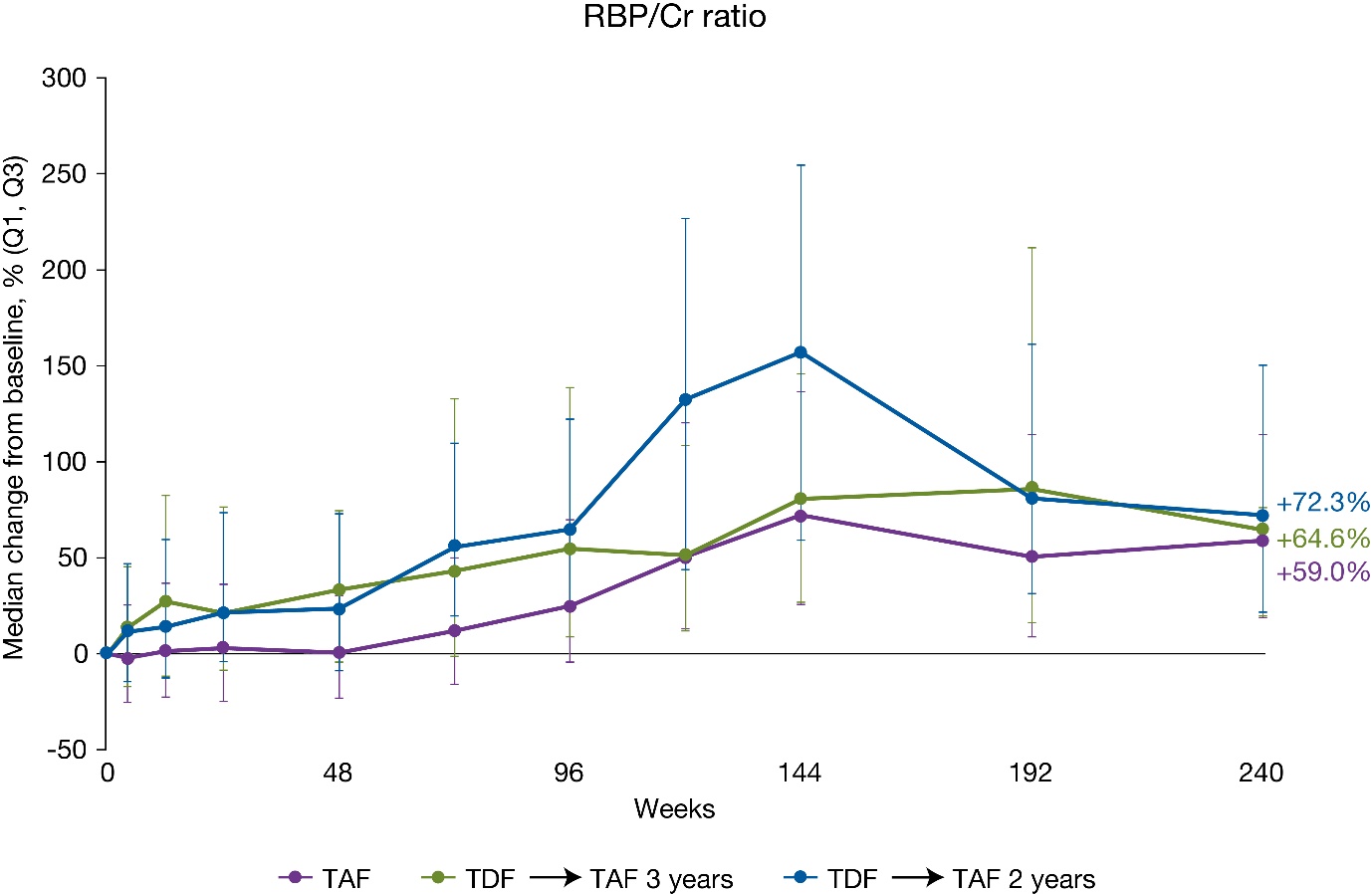
**

**Supplementary Figure S4. Changes in RBP/Cr ratios.** Median change from baseline in RBP/Cr ratio by study visit over 5 years. Cr, creatinine; Q, quartile; RBP, urine retinol-binding protein; TAF, tenofovir alafenamide; TDF, tenofovir disoproxil fumarate.
